# Evapotranspiration and crop coefficients of Italian zucchini cultivated with recycled paper as mulch

**DOI:** 10.1371/journal.pone.0232554

**Published:** 2020-05-06

**Authors:** Reginaldo Miranda de Oliveira, Fernando França da Cunha, Gustavo Henrique da Silva, Lucas Maltoni Andrade, Caio Vieira de Morais, Pedro Maurício Oliveira Ferreira, Flávio Pereira Gomes Raimundi, Agnaldo Roberto de Jesus Freitas, Caetano Marciano de Souza, Rubens Alves de Oliveira

**Affiliations:** 1 Department of Agricultural Engineering, Center of Agricultural Sciences, Federal University of Viçosa, Viçosa-MG, Brazil; 2 Department of Agronomy, Center of Agricultural Sciences, Federal University of Viçosa, Viçosa-MG, Brazil; Hellenic Agricultural Organization - Demeter, GREECE

## Abstract

Recycled paper has the potential to be used as a mulch for vegetable production and can be adopted for the cultivation of Italian zucchini. However, there have been no studies about the water savings or crop coefficient values used in irrigation management in this system; therefore, there is a need for more research. In view of the above, this study aimed to evaluate the effects of recycled paper mulch on evaporation and evapotranspiration in Italian zucchini and to determine the crop coefficients in its developmental stages. The study was carried out in two cultivation cycles conducted at the Lysimetric Station in Viçosa, MG, Brazil. The experiments were installed in a randomized block design with four replicates. Four lysimeter cultivation treatments were applied: without mulch (C); with recycled paper as mulch (CP); with only recycled paper (P); and with Bahia grass (G). Irrigation and drainage measurements were performed daily to calculate the crop and reference evapotranspiration, and thus the crop coefficient (Kc) values. The following characteristics were evaluated: fruit yield, NDVI and water productivity. For the cultivation of Italian zucchini using paper as mulch, Kc values of 0.54, 0.77 and 0.44 and Kcb values of 0.15, 0.45 and 0.18 are recommended for the initial, intermediate and final stages, respectively. NDVI can be used to estimate the Kc values for Italian zucchini. The use of recycled paper as mulch reduces the water consumption of Italian zucchini.

## Introduction

Irrigation and mulching techniques, when employed together, allow high yield and profitability in vegetable cultivation [1, 2]. The use of irrigation promotes small fluctuations in soil moisture in the root zone and maintains soil moisture at a level that is close to field capacity [3, 4]. These factors intensify water evaporation from the soil, that is, they increase losses in the portion of water that does not participate in any physiological/metabolic processes in the plant.

Traditionally, to avoid water losses through evaporation, straw or simply the remains of decaying leaves have been used as mulch. However, because it shows better results for water savings and has several other advantages, polyethylene has become the most commonly used material for this purpose [5–7].

Polyethylene, due to its chemical constitution, is not biodegradable under natural conditions. Therefore, polyethylene film must be removed from the soil after the end of the crop cycle [2, 8, 9]. However, plastic residues may accumulate and result in unsustainable land use, in addition to causing environmental problems [8, 9]. The main problems that can occur are (i) obstruction of infiltration, percolation and translocation of water and nutrients in the soil; (ii) negative effects on germination and root growth; (iii) secondary salinization of the superficial soil layer; and (iv) formation of substances that are harmful to plants after polyethylene degradation, such as phthalate esters, di-(2-ethylhexyl) phthalate, aldehydes and ketones [8, 10].

In this context, there is a continuous search for biodegradable chemical components to constitute mulches. Currently, raw materials with great potential and high sustainability are renewable carbon sources such as starch, cellulose and vegetable oils. Studies related to biodegradable mulches are still emerging, but there are already studies showing that biodegradable mulches do not compromise crop yield [9, 11].

The literature reports several studies using different types of paper as mulch, but Kraft paper was the most used because of its durability [12]. When used as mulch, paper can reduce the soil temperature [13], control weeds [14, 15] and save water [16].

Although recycled paper has great potential as mulch due to its durability and mechanical strength, scientific research on the use of this material as mulch in agriculture is scarce. Studies using recycled paper as mulch are necessary to evaluate the reduction in water evaporation and obtain technical coefficients for agricultural crops cultivated in Brazil. This is very important for certain crops, such as the Italian zucchini.

Italian zucchini (*Cucurbita pepo* L.) belongs to the Cucurbitaceae family and has an erect growth habit, despite its herbaceous stem [17]. This crop is among the ten most economically important vegetables consumed and produced in Brazil [18]. The plants are monoecious, i.e., they have male and female flowers. Italian zucchini has a low capacity for competition with weeds [18], and farmers must perform weed control throughout the entire zucchini growth cycle. Therefore, the use of mulch can simultaneously reduce the cost of weed control and save water. In general, Italian zucchini can be cultivated under microsprinkler, sprinkler, furrow and drip irrigation systems, the last being the most common. However, information on the coefficients adopted for irrigation management using mulches is still emerging, which indicates the need for research.

One current option for estimating the crop coefficient (Kc) is the use of the vegetation index [19]. As the attribution of Kc values is directly related to the phenological cycle of the crop, studies suggest that the temporal profiles of the vegetation indices can be used [19–21].

Among the developed vegetation indices, the Normalized Difference Vegetation Index (NDVI) is the most used due to its sensitivity to the presence of pigments that participate in photosynthetic processes [22] as well as its simple application, which allows the quick and efficient detection of variations in vegetation [23]. The spectral signature of green and healthy vegetation shows a clear contrast between the visible and near-infrared regions, which are used by the NDVI index [24]. Thus, it is necessary to assess the possibility of using NDVI to estimate the Kc of Italian zucchini, which will constitute an interesting strategy for assisting in irrigation management.

In view of the above, this study aimed to evaluate the effects of recycled paper mulch on evaporation and evapotranspiration in Italian zucchini and to determine the crop coefficients at different zucchini developmental stages.

## Material and methods

### Experimental area

The study was carried out in the experimental field of the Lysimetric Station of the Agricultural Engineering Department (DEA) of the Federal University of Viçosa (UFV), in Viçosa, MG, Brazil, at 20° 46' 09.30" S, 42° 51' 44.75" W and 674 m above sea level. According to Köppen’s classification, the climate of the region is Cwa, a humid temperate climate with a dry winter and a hot summer.

The soil used was a *Latossolo Vermelho-Amarelo distrófico* (Oxisol) collected in the 0–0.5 m layer and pounded to break up clods. Its chemical and physical-hydraulic attributes are presented in Table 1.

**Table 1:** Chemical and physical attributes of the soil used in the drainage lysimeters. Viçosa-MG, DEA-UFV, 2017.

pH	P	K		S	B		Cu	Mn	Fe	Zn
^..^ H_2_O ^..^	^**....................................................................................**^ **mg dm**^**-3 ....................................................................................**^									
4.21	1.6	69		0.1	0.06		3.6	21.2	49.8	0.93
**OM**	**Ca**^**2+**^	**Mg**^**2+**^		**Al**^**3+**^	**H + Al**	**SB**	**t**	**T**	**V**	**P-rem**
^..^ dag kg^-1 ..^	^**.....................................................................**^ **cmol**_**c**_ **dm**^**-3 ....................................................................**^									^**..**^ **mg L**^**-1 ..**^
**Coarse sand**	**Fine sand**		**Silt**				**Clay**	**Field capacity**	**Field capacity**	**Bulk density**
^....................................................... ....^ % ^.........................................................^								^**............... ...**^ **cm cm**^**-3 .................**^		^**.....**^ **g cm**^**-3 .....**^
**15.8**	14.1		14.6				55.5	0.291	0.236	0.990

P, K, Cu, Mn, Fe, Zn extracted with Mehlich 1 extractant. Ca^2+^, Mg^2+^, Al^3+^ extracted with 1 mol L^-1^ KCl. H + Al extracted with 0.5 mol L^-1^ calcium acetate. SB—sum of exchangeable bases. t and T—effective and pH-7 cation exchange capacities, respectively. V—base saturation index.

### Experimental design

Two experiments were conducted at different times (2017 and 2018) in order to confirm the results. The experiments were installed in a randomized block design with four replicates. Four treatments were applied:

- C—cultivation of Italian zucchini in a lysimeter without mulch;
- CP—cultivation of Italian zucchini in a lysimeter using recycled paper as mulch;
- P—lysimeter only with recycled paper as mulch; and
- G—lysimeter cultivated with Bahia grass.

**Fig 1:**
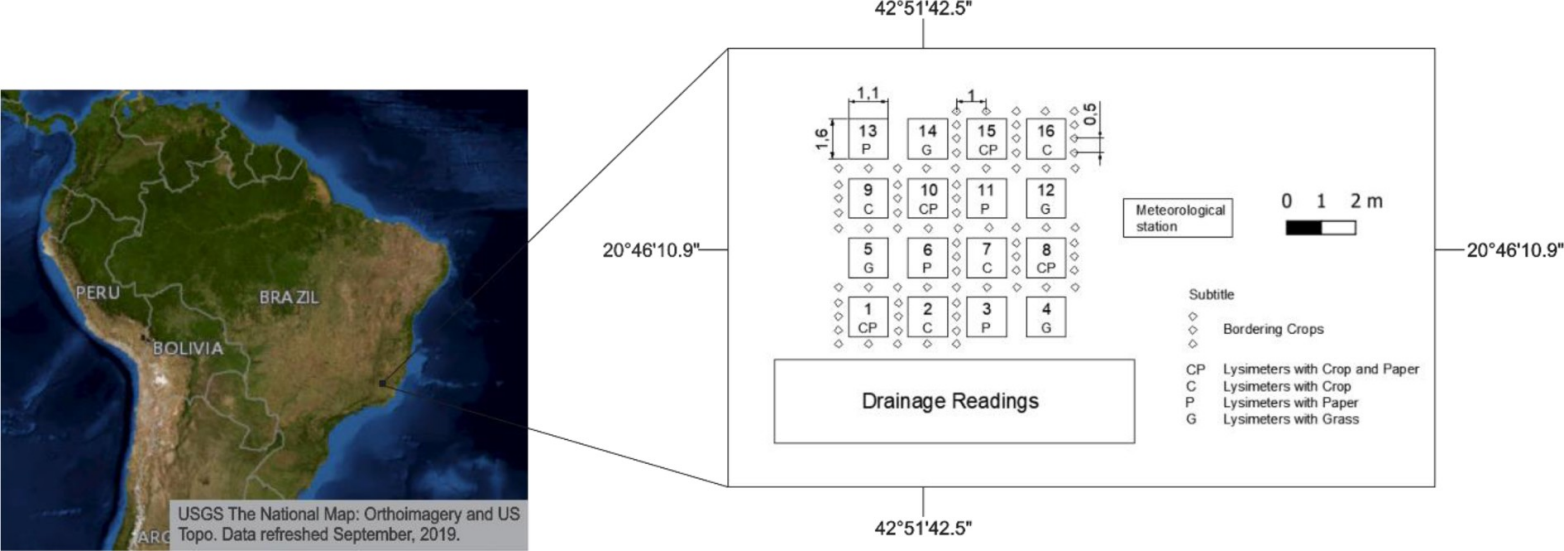
Sketch of the experimental area with the imposed treatments. Viçosa-MG, DEA-UFV, 2017–2018.

The experiments were set up in an 8.9-m-wide, 11.4-m-long (101.46 m^2^) experimental area where 16 drainage lysimeters are installed (Fig 1). The drainage lysimeters had 1.10 m width, 1.53 m length and 0.70 m depth, with 1.68 m^2^ of exposure area and 1.01 m^3^ of soil volume. The lysimeters were filled with 0.10 m of gravel, 0.15 m of sand and 0.40 m of soil. In addition, 0.05 m was left open above the soil level in the lysimeters to prevent water entry and exit through surface runoff.

### Management of lysimeters

Based on the chemical analysis (Table 1), soil acidity and fertility were corrected according to Trani et al. [25]. Lime was applied prior to transplanting at a dose of 3,000 kg ha^-1^. For fertilization, monoammonium phosphate (MAP) and potassium chloride (KCl) were applied at doses of 320 kg ha^-1^ and 70 kg ha^-1^, respectively.

The Italian zucchini (*Cucurbita pepo* L.) cultivar used was PX7051, from the Seminis^®^ company. The seedlings were produced in 128-cell expanded polystyrene trays with the substrate *Tropstrato HT Hortaliças*. The seeds were sown on 09/09/2017 and 03/08/2018, and the seedlings were transplanted on 09/29/2017 and 03/19/2018, when they had four true leaves. Italian zucchini was cultivated at a spacing of 1.0 m x 0.5 m, and each lysimeter had one row with three plants in the center and rows of plants as borders on the sides. Outside the lysimeters, there were also rows of plants and irrigation to minimize the bouquet and oasis effects. The last harvests of Italian zucchini occurred on 11/28/2017 and 05/18/2018, resulting in cycle durations of 61 days.

In the treatment in which Italian zucchini was cultivated using recycled paper as mulch, 10-cm-diameter holes were made for the emergence and growth of the stem. The recycled paper used was 40 μm thick and covered 100% of the soil in the lysimeters.

In the treatment using only a covering of recycled paper, there was no crop, but the same 10-cm-diameter holes were made. In the treatment with grass, the species Bahia grass (*Paspalum notatum*) was used and was maintained at heights between 10 and 15 cm.

Weeds were controlled by means of manual weeding, performed every day. The insecticide Decis^®^ (Bayer) and the fungicide Folicur^®^ (Bayer) at concentrations of 0.3 and 1.0 mL L^-1^, respectively, were also sprayed at two-week intervals to prevent the attack of pests, such as aphids and black cutworm, and fungal diseases, such as mildew.

Before starting each cycle, the soil in the lysimeters was saturated to bring the soil moisture to field capacity. After this, watering with a watering can was performed daily for the entire cycle. The water volume applied to each lysimeter was determined according to Eq 1. Excess irrigation (20% greater than the evaporation or evapotranspiration of the previous day) was performed to cause drainage in the lysimeters.

V=1.2LA (1)

where V is the water volume, L; L is the water depth applied, mm; and A is the lysimeter area, m^2^.

Drainage measurements were performed daily to allow the calculation of daily values of evaporation or crop evapotranspiration. The drainage water was collected in buckets inside a shelter through a PVC pipe with ½” diameter installed at the bottom of each lysimeter. Subsequently, the volume was obtained based on the mass of water measured on a precision scale.

Lysimetry is the most accurate method for the direct determination of evapotranspiration by soil water balance [26, 27]. The operation of the drainage lysimeter is based on the principle of conservation of water mass in a soil volume. In this volume, the water input (rain + irrigation) equals the water output (drainage + evapotranspiration). The parameters rain, irrigation and drainage are measured, and evapotranspiration is obtained by calculating the difference.

It is worth noting that all the percolate collected from the day in each lysimeter, was reapplied together with irrigation water in that same lysimeter to ensure the balance of salts and nutrients in the soil. However, before the water was applied to the lysimeters, the electrical conductivity and the pH of the water were measured to verify whether it would affect the crop.

The plants cultivated outside the lysimeters were irrigated by a drip irrigation system, and the water application amount was 100% of the crop evapotranspiration amount.

### Meteorological data

**Fig 2:**
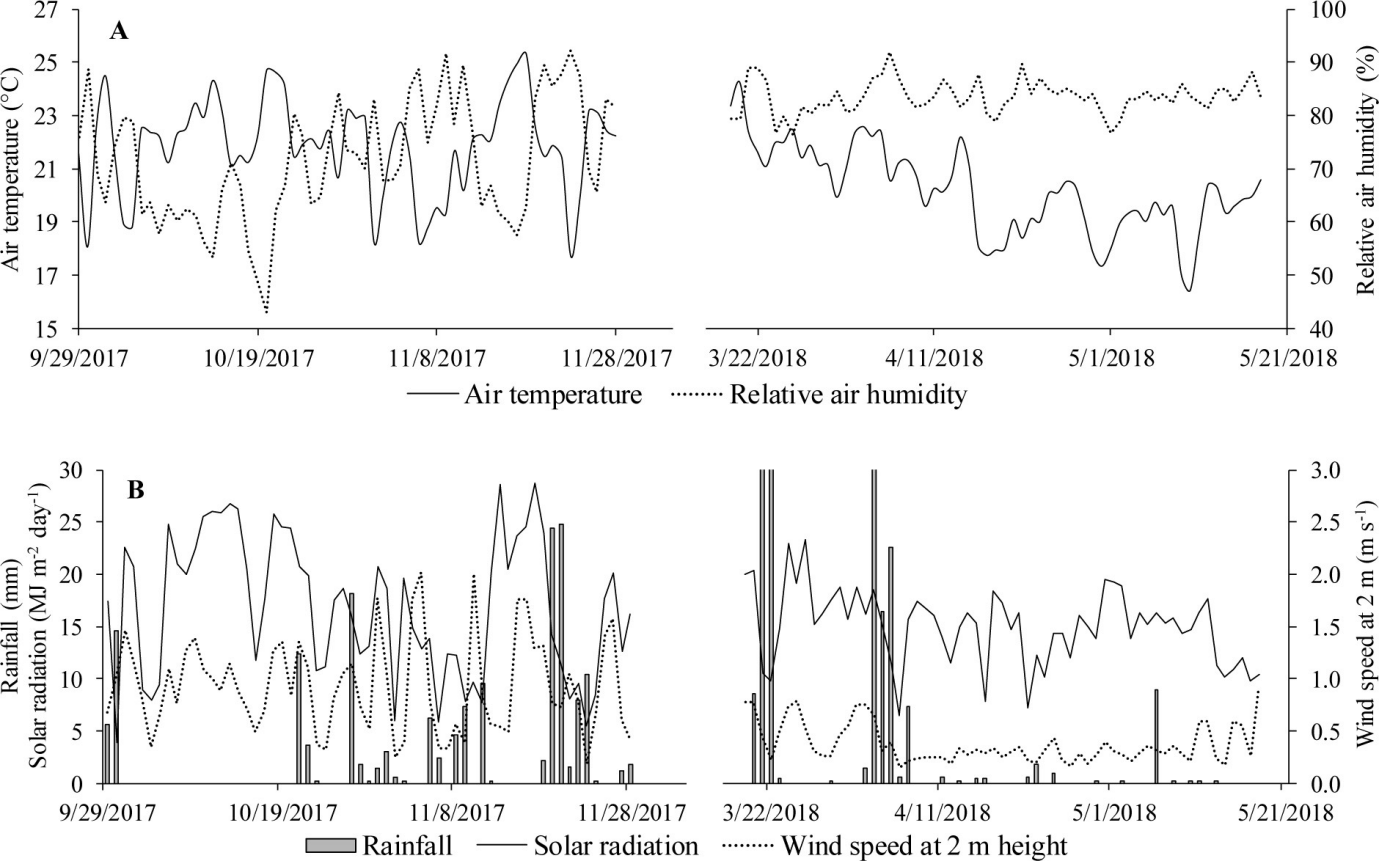
Daily variations in (A) air temperature (°C), relative humidity (%), (B) solar radiation (MJ m^-2^ day^-1^), wind speed at 2 m height (m s^-1^) and rainfall (mm) in both cultivation cycles of Italian zucchini. Viçosa-MG, DEA-UFV, 2017–2018.

Hourly data were collected for air temperature, relative humidity, solar radiation, wind speed and rainfall (Fig 2). These data were collected using an automatic meteorological station (DAVIS, Vantage Pro II model) installed in the area. Meteorological data were necessary to estimate the reference evapotranspiration (ETo) and to calculate the degree days (DD) in the cultivation cycles of Italian zucchini. The lower and upper basal temperatures were considered to be 8°C and 25°C, respectively. The methodology for DD calculation followed the recommendations of Scarpare et al. [28].

### Evapotranspiration

#### Reference evapotranspiration (ETo)

The hourly reference evapotranspiration (ETo) was estimated using the Penman-Monteith method [29], according to Eq 2. The daily ETo was obtained by integrating the hourly values of ETo over the 24-h period.

$$ETo=\frac{0.408\Delta (Rn-G)+\gamma \frac{37}{T+273}U_{2}(e_{s}-e_{a})}{\Delta +\gamma (1+0.34U_{2})}$$ (2)

where ETo is the reference evapotranspiration, mm h^-1^; Δ is the slope of the saturation vapor pressure curve, kPa°C^-1^; Rn is the net radiation on the surface, MJ m^-2^ h^-1^; G is the soil heat flux density, MJ m^-2^ h^-1^; T is the average daily air temperature,°C; U_2_ is the wind speed at 2 m height, m s^-1^; e_s_ is the saturation vapor pressure, kPa; e_a_ is the partial vapor pressure, kPa; and γ is the psychrometric coefficient, kPa°C^-1^.

#### Crop evapotranspiration (ETc)

The crop evapotranspiration (ETc) was determined from the water balance, which is based on the mass conservation law as presented in Eq 3 [30]. To avoid large variations in water storage in the soil (Δh) and maintain a soil moisture content close to field capacity, irrigation was performed daily at 8:00 a.m.

Thus, the term Δh in the mass conservation equation becomes very small and is disregarded in the water balance. Given these considerations, the equation for ETc determination was thus simplified to Eq 4.

P+I–D–ETc=±Δh (3)

ETc=P+I−D (4)

where P is the precipitation, mm; I is the irrigation depth, mm; D is the deep drainage, mm; ETc is the crop evapotranspiration, mm; and Δh is the variation in soil water storage, mm.

#### Single-crop coefficient (Kc)

The Italian zucchini crop coefficients (Kc) were calculated daily using Eq 5 [29].

$$Kc=\frac{ETc}{ETo}$$ (5)

where Kc is the dimensionless crop coefficient; ETc is the crop evapotranspiration, mm; and ETo is the reference evapotranspiration, mm.

The ETo was the ET obtained in the lysimeters cultivated with Bahia grass; however, this ETo was compared with the one obtained through Eq 2.

#### Dual crop coefficients (Kcb and Ke)

The soil evaporation coefficient (Ke) and the basal crop coefficient (Kcb) were determined daily using equations adapted from ALLEN et al. [29]. Ke was determined using Eq 6. For this, the evaporation obtained in the "P" treatment was divided by the evapotranspiration data obtained in the "G" treatment, as shown in Fig 1.

$$Ke=\frac{Ep}{ETo}$$ (6)

where Ke is the soil evaporation coefficient, dimensionless; Ep is the evaporation in non-vegetated lysimeters, mm; and ETo is the reference evapotranspiration, mm.

Because irrigation occurred daily, with a high irrigation frequency (one-day interval), it was assumed that the value of the stress coefficient (Ks) was equal to 1 (one). Consequently, Kcb was obtained from the difference between the crop and soil evaporation coefficients (Eq 7).

$$Kcb=\frac{Kc-Ke}{Ks}$$ (7)

where Kcb is the basal crop coefficient, dimensionless; Kc is the crop coefficient, dimensionless; Ke is the soil evaporation coefficient, dimensionless; and Ks is the stress coefficient, dimensionless.

### Evaluated characteristics

#### Recycled paper

Evaporation and permeability tests were conducted with the recycled paper. For the evaporation test, 54 pairs of containers with a cross-sectional area of 50 cm^2^ were partially filled with 12 mm water. For each pair of containers, one received a recycled paper covering, and in the other, the water was exposed to the environment. These pairs of containers were distributed in different environments and for different time intervals for the water inside to evaporate. After 48 hours, these containers were weighed on a precision scale, and the water lost was converted to the evaporated water depth (Eq 8). The results were plotted in a scatter plot for comparison of the results.

$$EV=\frac{Mev}{0.1\;\gamma \;A}$$ (8)

where EV is the evaporation of the water in the container, mm; Mev is the mass of water lost in the container by evaporation, g; γ is the specific mass of water, 1 g cm^-3^; and A is the cross-sectional area of the container, cm^2^.

For the permeability test, 29 trays of 40 cm diameter were covered with recycled paper, and 1.5 cm water was added. After 24 hours, all the water that passed through the recycled paper was collected and weighed on a precision scale. This mass of water was converted to volume and subsequently to water depth (Eq 9), and the distribution of the results was presented using a boxplot.

$$Pp=\frac{Mi}{0.1\;\gamma \;A\;T}$$ (9)

where Pp is the permeability of the recycled paper, mm d^-1^; Mi is the mass of water infiltrated into the recycled paper, g; γ is the specific mass of water, 1 g cm^-3^; A is the cross-sectional area of the container, cm^2^; and T is the water infiltration time, days.

#### Italian zucchini crop

The spectral data for the Italian zucchini were collected with an active sensor (GreenSeeker^®^ Handheld Crop Sensor, Trimble). It is a portable sensor with two types of light-emitting diode (LED) that emit active radiation at two wavelengths centered on red (660 nm) and near-infrared (770 nm), with a bandwidth of approximately 25 nm. The output data include five vegetation indices, but for the present study, only NDVI data were used. The data were collected dynamically at a distance of 0.8 to 1.2 m between the sensor and the target at three-day intervals.

At harvest, the yield of Italian zucchini fruits was evaluated. The mass measurements were obtained with a precision scale. Water productivity (WP) was obtained as the relationship between fruit yield and the volume of water evapotranspired by the crop [31], expressed in kg m^-3^ with Eq 10.

$$WP=\frac{Y}{\sum ETc}$$ (10)

where WP is the water productivity, kg m^-3^; Y is the fruit yield, kg m^-2^; and ∑ETc is the total volume of evapotranspired water, m^3^ m^-2^.

### Statistical analysis

For the agronomic characteristics of the Italian zucchini, individual statistical analyses were performed for each crop cycle. The data were subjected to the F test of the analysis of variance (ANOVA). Subsequently, the means were compared by the Tukey test at the 0.05 probability level.

Simple linear regression models (Eq 11) were fitted to formulate an equation that is capable of estimating the crop coefficient (Kc) with the NDVI values. The models were evaluated based on the significance of the regression coefficients using a t test at 0.05 probability and the coefficient of determination (R2 = S.S. Regression/S.S. Treatment).

$$y=a_{0}+b_{i}x_{i}$$ (11)

where y is the dependent variable, Kc; x_i_ is the independent variable, average NDVI; and a_0_ and b_i_ are the fitting coefficients of the linear model.

A paired-data t-test was used for NDVI data. The statistical analyses were carried out in R [32].

## Results and discussion

### Water consumption

**Fig 3:**
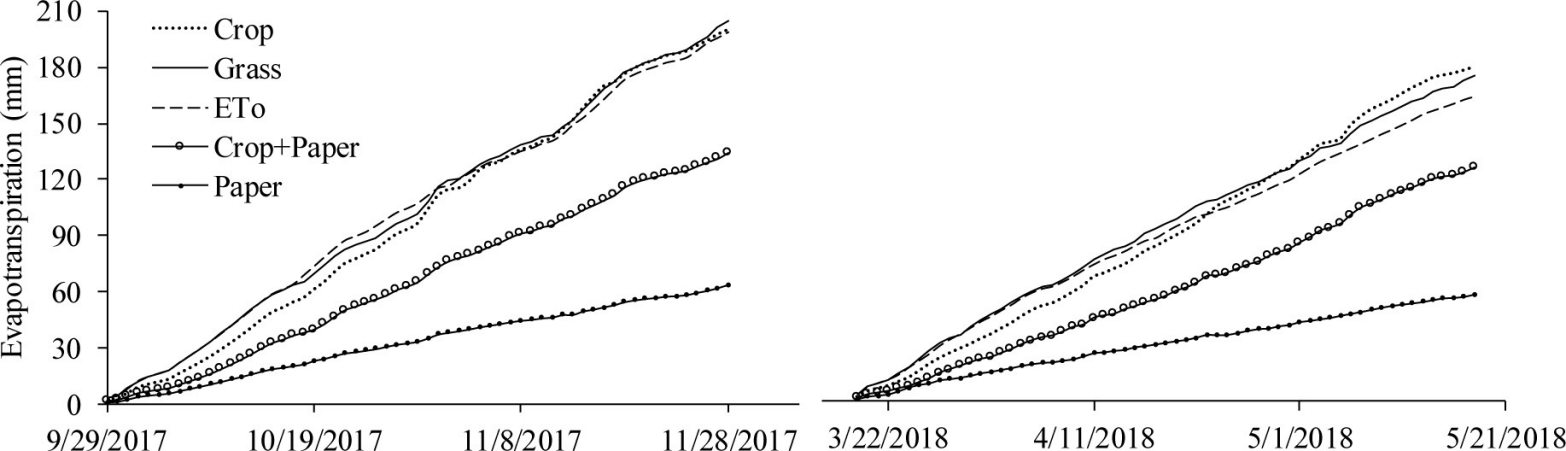
Evolution of water consumption in the drainage lysimeters with Bahia grass (Grass), recycled paper (Paper), Italian zucchini cultivated conventionally (Crop) and with mulch (Crop+Paper) and the reference evapotranspiration estimated by the Penman-Monteith method (ETo) for the two cultivation cycles. Viçosa-MG, DEA-UFV, 2017–2018.

Fig 3 shows the evolution of water consumption in the drainage lysimeters with Bahia grass, recycled paper, Italian zucchini cultivated conventionally and with mulch, and the reference evapotranspiration (ETo) estimated by the Penman-Monteith method [29] in the two cultivation cycles. In general, higher evapotranspiration rates were observed in 2017 due to the higher air temperatures (by 8%), wind speed (by 59%), and solar radiation (by 11%) and lower relative humidity (by 14%) than in 2018 (Fig 2).

Despite showing similar trends as the estimated ETo, the evapotranspiration from the Bahia grass measured in the drainage lysimeters was 3.2% and 6.7% higher than the ETo estimated by the Penman-Monteith model in 2017 and 2018, respectively. The small difference was possibly due to the “oasis effect”, as the experimental site was surrounded by an area that did not receive irrigation. The oasis effect is a phenomenon of advection, in which there is lateral (horizontal) transport of sensible heat from a dry area to a humid area. The extra sensible heat that reaches the humid area contributes to the increase in evapotranspiration [7, 33]. This observed and explained trend also reinforces the decision to use grass evapotranspiration, rather than the Penman-Monteith ETo, to estimate the crop coefficients of Italian zucchini.

Fig 3 also shows that the use of recycled paper as mulch reduced the water consumption of Italian zucchini plants in both cultivation cycles. These reductions were 32.7% and 29.6% in the cultivation cycles of 2017 and 2018, respectively. This reduction occurred because the recycled paper serves as a physical barrier and reduces soil water evaporation. The water savings promoted by the paper covering are directly proportional to the frequency of rain and/or irrigation. A high rain or irrigation frequency promotes small fluctuations in soil moisture in the root zone and maintains the soil moisture at close to field capacity [3, 4]. These factors intensify water evaporation from the soil, i.e., they increase the loss of the portion of water that does not participate in any physiological/metabolic process in the plant.

In addition, recycled paper can also promote a reduction in soil temperature [9, 34], decreasing the accumulation of sensible heat and consequently its conversion to latent heat for soil water evaporation. Thus, the use of recycled paper as mulch to minimize evaporative water losses in the soil is justified. Since the recycled paper is only slightly permeable, its presence results in low effective precipitation. On the other hand, the paper covering also prevents excess water accumulation in heavy soils and nutrient leaching when excessive rain occurs.

Despite reducing the evapotranspiration of Italian zucchini, the recycled paper did not completely prevent the loss of soil water through evaporation. Fig 3 shows that the accumulated evaporations in the lysimeters with recycled paper were equivalent to 63.8 mm and 59.3 mm in the cycles of 2017 and 2018, respectively. Three openings of 10 cm diameter were made in each lysimeter, as in the lysimeters with Italian zucchini plants. These openings in the recycled paper were made at the same time as the transplanting of the seedlings. Thus, the area not covered by the recycled paper in each lysimeter was 235.6 cm^2^, i.e., 1.34% of the lysimeter surface area was exposed soil.

**Fig 4:**
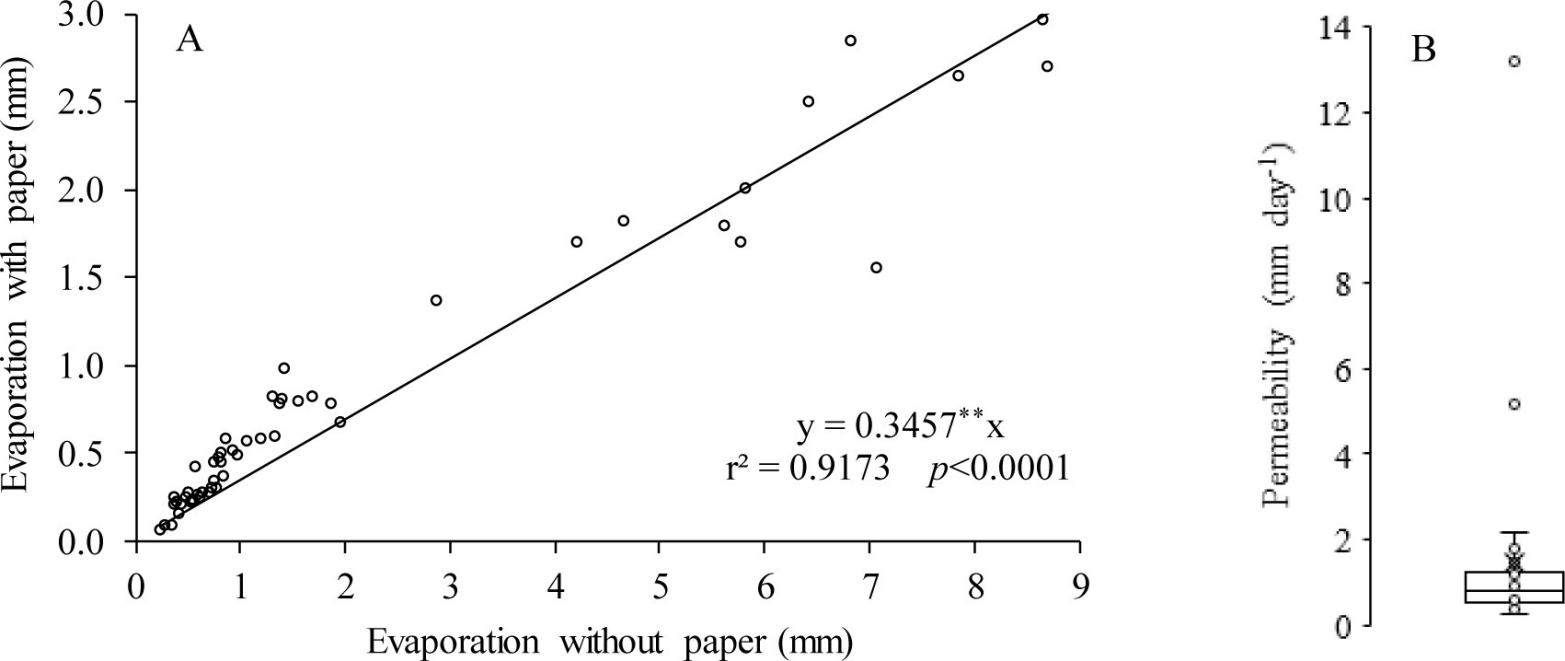
Evaporation occurring in containers with and without recycled paper coverings (A) and the permeability of the recycled paper (B). Viçosa-MG, DEA-UFV, 2019. **p < 0.0001.

**Fig 5:**
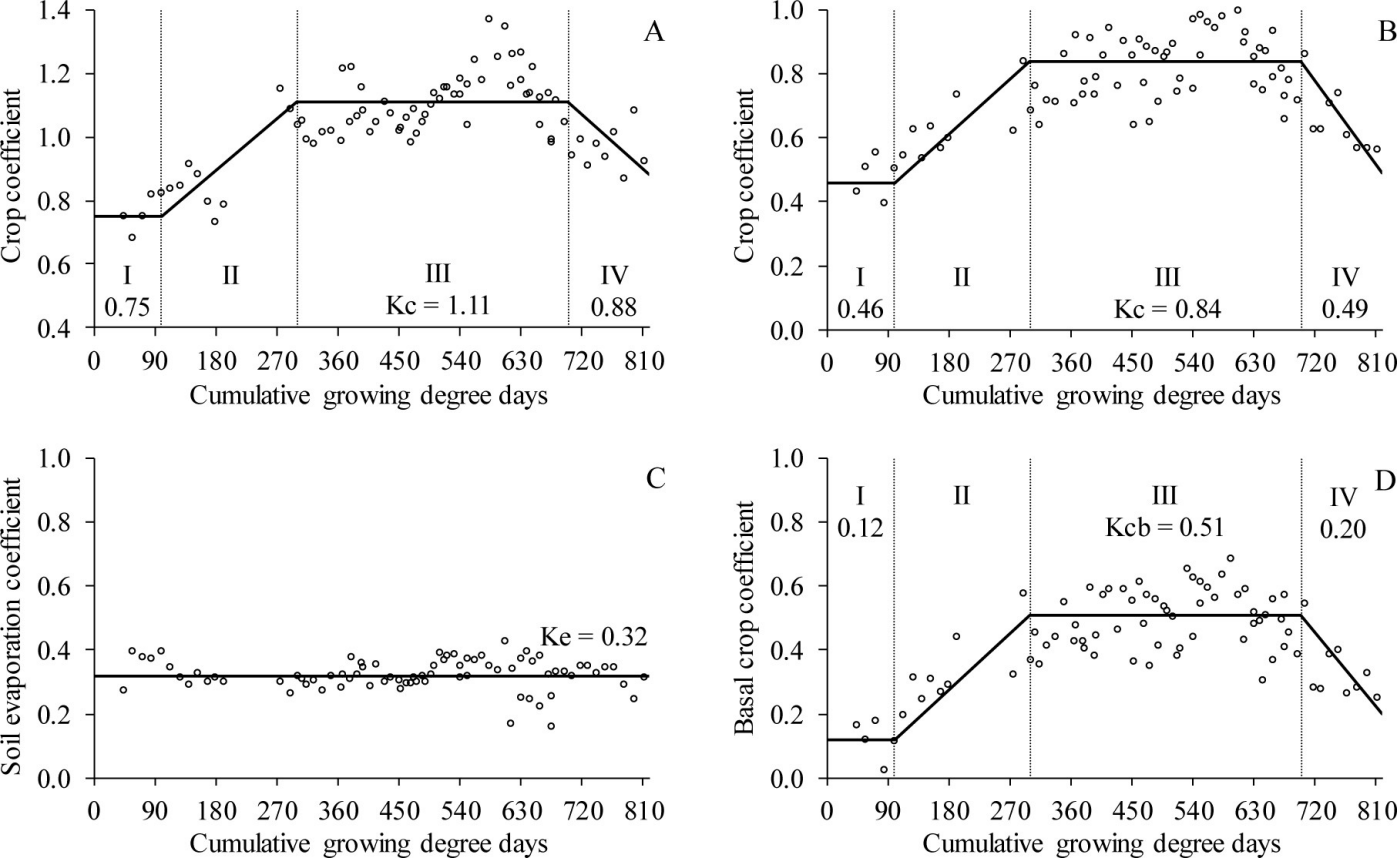
Crop coefficient (Kc) of Italian zucchini (A) cultivated without mulch and crop coefficient—Kc (B), evaporation coefficient—Ke (C) and basal crop coefficient—Kcb (D) of Italian zucchini cultivated with recycled paper as mulch. I, II, III, IV correspond to the development stages of the Italian zucchini. Viçosa-MG, DEA-UFV, 2017–2018.

The evaporation tests in containers with and without recycled paper cover corroborate the results obtained in the lysimeters (Fig 4A). According to the regression equation, the recycled paper was responsible for the 65.43% reduction in evaporation from the evaluated containers. Notably, in addition to providing the advantage of reducing evaporation, the recycled paper also allows part of the rainfall to be used by the crop. Fig 5B shows that the permeability of the recycled paper was equal to 1.46 mm day^-1^. Thus, the use of recycled paper as mulch is expected to allow water replacement at lower soil depths, thus promoting energy and electricity savings.

### Crop coefficients

Italian zucchini cultivated in the conventional system showed average crop coefficient (Kc) values of 0.75, 1.11 and 0.88 in its initial, intermediate and final stages, respectively (Fig 5A). For a local variety in Turkey, Yavuz et al. [35] recommend values of 0.56, 0.95 and 0.65 for the same stages, respectively. It is possible that the Kc values obtained in the present study were higher because the Italian zucchini crop received irrigation and/or precipitation daily, whereas the above-mentioned study used a 7-day irrigation interval. According to Allen et al. [36] and Shrestha and Shukla [37], the higher the frequency of rainfall or irrigation, the higher the Kc.

Allen et al. [29] also reported more moderate values of Kc for the zucchini crop. These authors recommend values of 0.50, 0.95 and 0.75 for the initial, intermediate and final stages, respectively. However, these values are recommended for conditions in which the minimum relative air humidity is above 45%. Thus, the Kc values obtained in the present study were higher because the minimum relative air humidity remained below the mentioned value for 31% of the experimental period. According to Shrestha and Shukla [37], higher Kc values are expected with the reduction of the ambient relative humidity.

For Italian zucchini cultivated with recycled paper as mulch, the Kc values were 0.54, 0.77 and 0.44 in the initial, intermediate and final stages, respectively (Fig 5B). The recycled paper led to reductions of 39%, 24% and 44% in the Kc values for the initial, intermediate and final stages, respectively. The larger contributions to the reduction of Kc observed in the initial and final stages are due to soil exposure and the loss of vegetative vigor, respectively.

At the beginning of conventional cultivation cycles, water evaporation from the soil contributes more to the process of evapotranspiration [38]. This is due to the small leaf area, in addition to resulting in lower transpiration rates, also contributes to greater soil exposure. With the soil exposed to direct solar radiation, a higher gain of sensible heat is expected; the sensible heat is converted to latent heat, resulting in higher evaporation rates. Thus, the physical barrier imposed by the recycled paper is more efficient in reducing evaporation at the beginning of the cultivation cycle. In the intermediate stages, the crop itself protects the soil from direct radiation.

In the final stage, the recycled paper mulch led to greater reductions in Kc due to the loss of crop vigor. Thus, the transpiration component was also reduced, and evaporation began to govern the process of evapotranspiration, triggering the same effects described for the beginning of the cycle. Another possible explanation is that the Italian zucchini cultivated with mulch exhibited an accelerated growth cycle [11, 18], which would also contribute to the reduction in Kc values.

The soil contained in the lysimeters with recycled paper showed evaporation corresponding to 32% of the grass evapotranspiration (Fig 5C). The values of the basal crop coefficient (Kcb) are shown in Fig 5D. Kcb values can be used, if needed, when the evaporation coefficient (Ke) is obtained by estimation.

### Kc estimation by NDVI

The NDVI values of lysimeters with Italian zucchini cultivated with and without recycled paper as mulch did not differ throughout the cultivation period according to the paired-data t-test (*p-value* = 0.3580). This may have occurred because the recycled paper and the soil in the lysimeters have similar values of reflectance at the red and near-infrared wavelengths. In the first reading performed in the cultivated lysimeters, soon after the Italian zucchini was transplanted, the mean NDVI values were 0.17±0.01 and 0.18±0.01 in the treatments with and without recycled paper, respectively. The lysimeters with only recycled paper had a mean NDVI value of 0.15±0.00 in all evaluations. Finally, the lysimeters with Bahia grass showed a mean NDVI of 0.73±0.10; the high value of the standard deviation is due to the trimming performed to maintain the height of the grass between 10 and 15 cm.

**Fig 6:**
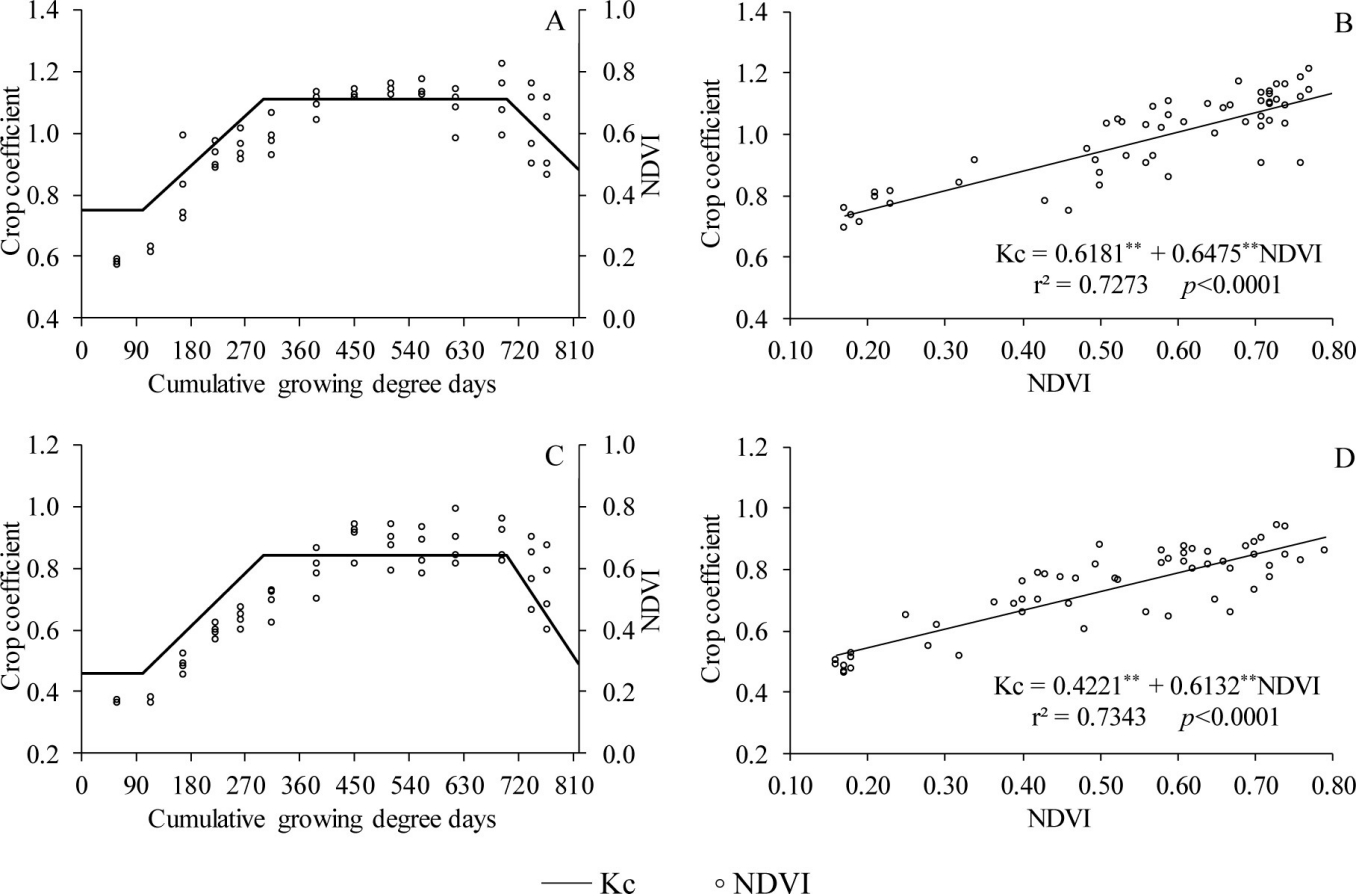
Relationships between the crop coefficients and the normalized difference vegetation index (NDVI) of Italian zucchini in the conventional system (A and B) and cultivated with recycled paper as mulch (C and D). Viçosa-MG, DEA-UFV, 2017–2018. **p < 0.0001.

According to Alface et al. [19], the rapid and accurate estimation of Kc can be performed by measures of the normalized difference vegetation index (NDVI). The behavior of the NDVI of Italian zucchini cultivated conventionally and using recycled paper as mulch is shown in Fig 6A and 6C, respectively. The Kc and NDVI profile curves showed similar trends.

Fig 6B and 6D show the equations fitted by the relationship between NDVI and Kc. Approximately 73% of the variation in the NDVI value can be explained by the variation in the Kc of the Italian zucchini crop. The obtained *p-value* confirmed that the fit was significant.

This analysis was also conducted by Singh and Irmak [20], who found a high correlation when establishing the relationship between NDVI and Kc for corn and soybean crops in southern central Nebraska, USA. It is important to emphasize that if the mean values of NDVI were considered in this analysis, a better fit for the data would be obtained. Additionally, corroborating the results obtained in the present study, Esquerdo et al. [24] found similarity in the Kc and NDVI curves when analyzing the temporal profiles of NDVI generated from remote sensing data with the VGT-S10 product for soybean crops in Brazil.

Alface et al. [19] states that the spectral reflectance of agricultural crops can provide an indirect estimation of Kc values. This was confirmed by the Kc and NDVI curves in the present study. Therefore, the NDVI can be used as an indirect way to obtain the Kc for Italian zucchini cultivated conventionally or using recycled paper as mulch.

### Fruit yield and water productivity

The use of recycled paper as mulch did not affect any of the agronomic characteristics of Italian zucchini in the different cultivation cycles (Table 2). In contrast to the present study, Silva [18] found that paper cover promoted higher yields of Italian zucchini fruits in a study with different types of mulch. The author attributed this higher yield to the presence of weeds in the treatment without mulch (conventional system) since the recycled paper successfully suppressed weeds. In the current study, weeds were systematically and rigorously controlled because the objective was to measure the water consumption and crop coefficients of only the Italian zucchini. Therefore, weeds did not hamper their growth and development in any cultivation system.

**Table 2:** Mean values of crop evapotranspiration (ETc), water productivity (WP) and fruit yield of Italian zucchini cultivated in the conventional system (Crop) and with recycled paper as mulch (Crop+Paper). Viçosa-MG, DEA-UFV, 2017–2018.

Treatment	^....................... ....^ Cycle 2017 ^....................... ....^			^....................... ....^ Cycle 2018 ^....................... ....^		
	ETc	Yield	WP	ETc	Yield	WP
	(mm)	(kg m^-2^)	(kg m^-3^)	(mm)	(kg m^-2^)	(kg m^-3^)
Crop	199.6 ^a^	3.886	19.23	182.6 ^a^	0.665	3.646
Crop+Paper	134.3 ^b^	0.992	7.242	128.5 ^b^	0.951	7.303
*p* (F test)	0.024	0.102	0.101	0.014	0.382	0.149
CV(%)	13.04	72.09	59.07	9.39	48.27	14.87

There were high values of coefficient of variation in fruit yield for both cycles of Italian zucchini. A specific explanation for this problem was not found in the literature. In addition, Table 2 also shows a trend of reduction in Italian zucchini fruit yield in the second cycle. Such reduction may have occurred due to climatic factors, as Fig 1 indicates an increase in relative humidity and decrease in air temperature at the end of the 2018 cycle. Since daily irrigations were necessary to manage the lysimeters, this association between water and climate may have favored the occurrence of diseases. However, it is worth highlighting that these diseases did not reach a level of economic damage, since preventive fungicide applications were performed.

Recycled paper reduced the water consumption of Italian zucchini, but it was observed that for each treatment the water consumption was similar in both cycles. This similarity occurred because the climate conditions promoted similar ETo values in both cultivation cycles (Fig 3). Since Kc values were also similar, the values of water consumption in the different treatments were also expected to be similar in both cultivation cycles.

Although the recycled paper led to lower water consumption by Italian zucchini in the different cultivation cycles, its water productivity was not affected by the cultivation system (Table 2). Water consumption is the denominator in the calculation of water productivity. However, the numerator, fruit yield, was the determinant for this result, because fruit yield showed high coefficients of variation.

## Conclusions

Recycled paper has potential to be used as mulch in Italian zucchini cultivation. However, there is no information on water saving and crop coefficients, which generates a demand for research. Thus, we evaluated the effect of recycled paper as mulch on evaporation and evapotranspiration of Italian zucchini and determined the crop coefficients in its development stages.

We conclude that the use of recycled paper as mulch reduces the water consumption of Italian zucchini. For the cultivation of Italian zucchini using paper as mulch, crop coefficient values of 0.54, 0.77 and 0.44 and basal crop coefficient values of 0.15, 0.45 and 0.18 are recommended for the initial, intermediate and final development stages, respectively. The Normalized Difference Vegetation Index (NDVI) can be used to estimate the crop coefficient values of Italian zucchini. The use of recycled paper as mulch in Italian zucchini cultivation does not alter its water productivity and fruit yield.

The present study is unprecedented, but future research should be conducted considering other vegetables and regions to consolidate the use of recycled paper as mulch. Despite that, the results found here a promising and can serve as the basis for future studies and help farmers and technicians in the irrigation management of Italian zucchini.

## Supporting information

S1 File (RAR)
